# Medication Literacy and Medication Self-Management: A Cross-Sectional Study in Hospitalised Patients (65+) With Polypharmacy

**DOI:** 10.1155/jonm/5430265

**Published:** 2024-12-31

**Authors:** Laura Mortelmans, Jenny Gentizon, Tinne Dilles

**Affiliations:** ^1^Department of Nursing Science and Midwifery, Centre for Research and Innovation in Care (CRIC), Nurse and Pharmaceutical Care (NuPhaC), Faculty of Medicine and Health Sciences, University of Antwerp, Antwerp, Belgium; ^2^Research Foundation Flanders (FWO), Brussels, Belgium; ^3^Institute of Higher Education and Research in Healthcare-IUFRS, University of Lausanne (UNIL), Lausanne University Hospital (CHUV), Lausanne, Switzerland

**Keywords:** hospitalisation, medication literacy, medication self-management, nursing, older adults, polypharmacy

## Abstract

This study aimed to examine the relationship between medication literacy and the degree to which patients are considered capable of medication self-management, the factors influencing the strength of this relationship and the factors influencing a patient's capability for medication self-management. Between January and April 2022, a cross-sectional survey was conducted on hospitalised patients (65+) with polypharmacy. Medication literacy was evaluated using the MEDication Literacy Assessment of Geriatric patients and informal caregivers (MED-fLAG). The SelfMED assessment determined a patient's capability for medication self-management. The relationship between MED-fLAG and SelfMED was explored with Pearson's *r*. Moderation analysis was used to explore the factors influencing the strength of the relationship between both. Linear regression explored the factors influencing SelfMED scores. In total, 169 patients participated in the study. Patients considered themselves more capable of in-hospital medication self-management (8/10 rating) compared to nurses' and physicians' opinions (6/10 rating). Interactive medication literacy scored higher (mean = 3.0) than functional (mean = 2.9) and critical medication literacy (mean = 2.8). The more medication literacy skills, the more patients were considered able for medication self-management by healthcare providers (*r* = 0.630, *p* < 0.001). Moderation analysis could not reveal any factors that significantly affected the strength of the relationship between both. Age, managing medication independently at home, the number of chronic conditions and medication literacy were significant predictors of a patient's capability for in-hospital medication self-management. This study demonstrated a rather strong correlation between the results of the MED-fLAG and the SelfMED assessment. Hence, SelfMED can be used as a stand-alone first screening instrument to determine a patient's capability for in-hospital medication self-management, without first assessing medication literacy. MED-fLAG can provide valuable insights into the medication literacy of patients considered less capable of managing their medication, allowing medication information and interventions to be tailored to the patient.

## 1. Introduction

In 2022, more than one-fifth (21.1%) of the population in Europe was aged 65 and over [1]. Global population ageing leads to a growing number of people affected by chronic diseases, which represents a major challenge to healthcare systems to meet the healthcare needs of these patients [2]. To relieve pressure on healthcare systems, patient empowerment and self-management, as part of self-care, are increasingly encouraged [3, 4].

Self-management of medication is a complex self-care activity requiring patients to have knowledge, skills and behaviours to manage a medication regimen as prescribed [5]. However, research showed that older patients often experience their medication self-management as difficult [6–8]. Elderly, in particular, are more likely to experience problems with medication self-management due to functional and cognitive decline [9] as well as pharmacokinetic and pharmacodynamic changes making them vulnerable to adverse events [10]. In addition, ageing increases the risk of polypharmacy (i.e., concurrent use of five medications or more) [11], which is associated with a higher number of medicines, a more complex medication regimen and drug-related problems such as side effects, interactions and medication errors [12]. Furthermore, older adults living with chronic conditions are more frequently hospitalised and exposed to medication changes during their hospital stay: Rather than being simplified, medication regimens are often made more complex, dosages are changed, and new medicines are initiated which in turn complicate medication self-management postdischarge [13, 14].

Research showed that medication self-management is worse in patients with low health literacy skills [15–19]. Medication literacy, as derived from health literacy, is indispensable for proper medication self-management. Medication literacy was recently defined as ‘the degree to which older adults can develop and maintain functional, interactive and critical skills' [20, 21]. These skills include, for example, the capacity to comprehend, prepare and self-administer medicines (functional skills), the capacity to actively engage with healthcare professionals, communicate concerns and participate in decision-making (interactive skills), as well as the capacity to seek reliable medication-related information, gain control over medication management and address problems appropriately (critical domain) [20]. To gain insight into the medication literacy of older adults, the MEDication Literacy Assessment of Geriatric patients and informal caregivers (the MED-fLAG) was recently developed, and its first measurement properties have been tested [20].

The literature already highlighted the importance of assessing an individual's capacity to manage medication [22, 23], but the formal assessment of medication self-management is not routinely performed in clinical practice [23]. Insight into a patient's ability to manage medications whilst in hospital can, however, be used as a resource to tailor interventions to the patient's needs (e.g., arranging help with medication use at home) to enhance the safe and correct use of medication postdischarge. The SelfMED assessment, a stepped assessment performed by a nurse, the patient and the treating physician, has been used to determine the extent to which patients are considered capable of medication self-management, whilst in hospital [24]. The SelfMED assessment is a very short and easy scale based on the perception of the patient, nurse and physician while MED-fLAG is a more comprehensive scale, developed to get an in-depth understanding of medication literacy and all its domains.

It remains uncertain whether evaluating medication literacy would contribute to the evaluation of older hospitalised patients' eligibility for self-managing their medication. Nevertheless, our working hypothesis posits a potential relationship between the level of medication literacy, measured by the MED-fLAG and the extent to which a patient can be considered competent in managing their medication independently based on the SelfMED assessment.

The primary objective was to examine the relationship between the medication literacy and the extent to which older patients with polypharmacy (65+) are considered capable of medication self-management during hospital stay.

The secondary objectives were as follows:

1. to examine the influence of sociodemographic and clinical factors on the relationship between medication literacy and medication self-management.
2. to examine the influence of sociodemographic and clinical factors on the extent to which patients are considered capable of medication self-management.

## 2. Methods

### 2.1. Design

A multicentre quantitative, cross-sectional survey study was conducted on hospitalised patients (65+) with polypharmacy.

### 2.2. Setting, Participants and Sample Size

Patients were recruited from an internal medicine ward, a cardiology ward, two oncological wards, a geriatric ward and a surgical ward at two hospitals in the Netherlands. All eligible patients admitted to the participating wards between January and April 2022 and willing to participate were included (i.e., consecutive sampling). Eligibility was considered by the (head)nurse based on the following criteria: being at least 65 years old, polypharmacy at hospital admission (i.e., daily intake of at least 5 chronic medicines) and speaking Dutch. Patients with a neuropsychiatric diagnosis of dementia and patients incapable of providing consent to participate in the study were excluded.

Power calculation showed that, to detect a significant Pearson's correlation of at least *r* = 0.3 between the level of medication literacy and the patients' capacity to self-manage medication, with a power of 0.80, and a type I error rate of 0.05, a minimum sample of 84 patients would be needed. However, multiple regression requires higher sample sizes. According to the guideline by Tabachnick and Fidell, the sample size should be greater than 50, plus 8 times the number of predictors [25]. Considering 11 predictors (i.e., 11 sociodemographic factors), a sample size of at least 138 patients is needed.

### 2.3. Data Collection

After obtaining signed consent from the participants, they were included in the study. Patients refusing to participate were asked to fill out a nonresponse form. Patients were asked to complete a paper-based survey consisting of three parts: (1) self-developed questions on sociodemographic characteristics, (2) the MEDication Literacy Assessment of Geriatric patients and informal caregivers (MED-fLAG) to measure medication literacy and (3) the patients' self-assessment (SelfMED) to assess the own capacity to self-manage medication during hospitalisation. The nurse and the attending physician were asked to carry out an assessment of the patients' ability to manage their in-hospital medication, using the SelfMED instrument.

#### 2.3.1. Measurements

The following sociodemographic characteristics were collected to describe the research population: age, gender, highest level of education, living situation, help from others, type and reason for hospitalisation, length of hospital stay, number of chronic conditions and number of medicines per day. The European Qualifications Framework (EQF) was used to classify patient's level of education [26].

The MED-fLAG was used to evaluate the patient's medication literacy skills [20]. The MED-fLAG is a patient-reported outcome measure with evaluative application for prescribed and nonprescribed medicines, irrespective of the galenic form. The MED-fLAG consists of 56 items in three domains: (1) functional medication literacy (22 items), (2) interactive medication literacy (13 items) and (3) critical medication literacy (21 items) [20]. The scoring options are graded as follows: a) level of difficulty (Likert scale from 4 = not difficult at all to 1 = very difficult/impossible) and b) frequency of actions (Likert scale from 4 = always to 1 = never) [20]. Higher MED-fLAG scores indicate higher medication literacy skills.

The 56-item MED-fLAG was originally developed in the French language and tested for its content validity [20]. A proficient bilingual speaker translated from French to Dutch. The Dutch version was then back-translated by a native French speaker from the University of Antwerp's language institute, which led to minor wording adjustments. Subsequently, the Dutch translation of the MED-fLAG was used to collect data.

The SelfMED assessment is an assessment tool evaluating patients' eligibility for in-hospital medication self-management, originally developed in Dutch language [24]. The assessment has been validated by healthcare providers and through a multidisciplinary expert meeting. The questions of the nurse assessment were tested for inter-rater reliability [24]. Subsequently, the assessment was pilot-tested in a hospitalised patient population taking chronic medicines [27]. In the current study, the SelfMED assessment was used as originally developed and consists of three parts:

1. A nurse assessment: It is a ten-statement assessment based on the information obtained during the intake or information available in the patient's file. Nurses had to indicate whether they agreed or disagreed with the statements from the assessment and whether there was a contraindication for medication self-management. At the end of the assessment, the nurse formulated an advice for the treating physician about the patients' capability to self-manage medication (capable or not). Furthermore, the nurse indicated on a ten-point scale the extent to which the patient is capable.
2. A patient self-assessment: The questions in the assessment comprised current medication management at home (1 item), the patient's willingness to self-manage medication in hospital (1 item) and the patient's medication adherence at home (7 items). On a ten-point scale, participants had to indicate the extent to which they believe they are capable to self-manage their medicines during hospitalisation (1 = *absolutely not capable*, 10 = *fully capable*).
3. A physician assessment: The treating physician indicated if the patient was capable to self-manage medication during hospitalisation (yes/no) and the extent to which the patient was capable (ten-point scale). Both the nurse's assessment and the patient's assessment were available to make an informed decision.

Normally, the stepped SelfMED assessment is used to decide whether in-hospital medication self-management is allowed or not. However, this study was limited to performing the assessment and did not involve actual in-hospital medication self-management by the patient.

### 2.4. Data Analysis

The data collected were entered into Excel. Subsequently, data were analysed using IBM SPSS Statistics Version 28.0. For descriptive statistics, continuous variables (MED-fLAG scores, SelfMED scores, age, length of hospital stay, number of chronic conditions and number of medicines per day) were expressed as mean and standard deviation (SD) or median and range, depending on the distribution. The categorical variables (i.e., gender, level of education, living situation, help from others, type and reason for hospitalisation, assessment of medication self-management by patients and nurses using statements) were expressed in relative and absolute frequencies. Normality of the distribution was assessed using *Z*-scores [28].

Utilising the Pearson r test, inferential statistics were applied to explore the relationship between medication literacy scores and the extent to which patients were considered capable of medication self-management during hospital stay. Using moderator analysis, the factors influencing the strength of the relationship between MED-fLAG and SelfMED were explored. Sociodemographic characteristics were considered as potential moderator variables. Standardised values were created. Subsequently, interaction terms were calculated by computing the product between the independent (MED-fLAG) and the moderator variable. Lastly, a linear regression analysis was conducted to test the interaction effect.

Using linear regression analysis, the factors associated with the extent to which patients are considered capable of medication self-management (dependent variable) were studied. Patients' medication self-management capacity here refers to the mean score of the physician's and nurse's rating, considering this score is likely to be more objective than the patient's score. Sociodemographic characteristics of the respondent and medication management-related factors (i.e., number of medicines, medication management at home) were considered. In preparation of the regression analysis, the relationship between these factors and the dependent variable was described using independent t-test and Spearman's rho correlations. Afterwards, a univariate analysis was performed to determine which factors were significant predictors of the extent to which patients are considered capable of in-hospital medication self-management. Subsequently, only significant variables from the univariate analysis were included in the multiple linear regression using enter-method. A two-sided *p* value < 0.05 was considered significant.

### 2.5. Ethics

This study was conducted in accordance with the Declaration of Helsinki [29]. All individuals signed an informed consent form. The research protocol was approved by the ethics committee of both participating hospitals (ID 2021.075 and ID 2021-3023).

## 3. Results

### 3.1. Characteristics of the Research Population

Of the 193 patients invited to participate in the study, 169 completed the survey (response rate 87.6%). Reasons for nonparticipation were as follows: too sick/too tired (*n* = 6; 25%), not willing to share personal data (*n* = 4; 16.7%), not willing to spend time on research (*n* = 3; 12.5%), research is too difficult (*n* = 3; 12.5%), not interested in participating in research (*n* = 2; 8.3%), participating is too much of an effort (*n* = 1; 4.2%) and not willing to sign informed consent (*n* = 1; 4.2%). Three patients did not want to provide a reason for nonresponse.

Participants' mean age was 76 years, with 57% being male and 49% having attained an EQF educational level of 5 or 6. Most of the participants (69%) were admitted to the hospital without prior planning, and roughly half had hospital stays lasting 2 to 3 days. The main reason for hospitalisation was treatment (50%) followed by observation (17%). Only 6% were admitted due to medication review. Most participants (76%) indicated that they take 5 to 10 medicines each day. Participants' characteristics are shown in Table 1.

### 3.2. Medication Literacy Scores

Patients scored on average 2.9 (SD = 0.69) out of a four-point Likert scale on the MED-fLAG. The interactive domain scored highest (*M* = 3.0, SD = 0.79), followed by the functional (*M* = 2.9, SD = 0.76) and critical domains of medication literacy (*M* = 2.8, SD = 0.68). Analysis of the individual items included within the MED-fLAG instrument can be found in Supporting Table S1.

### 3.3. Patients' Capacity to Self-Manage Medication

The results of the patients' self-assessment are shown in Table 2. The majority of participants (67%) self-managed their medication completely independently at home. About 58% indicated their willingness to self-manage medication during hospitalisation. Patients rated their ability to self-manage medication in-hospital on average 8/10.

**Figure 1:**
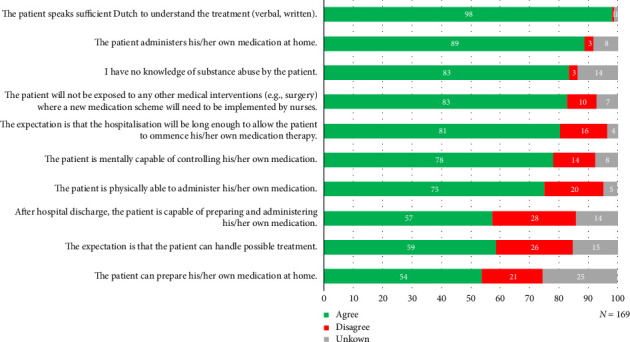
Nurse assessment regarding medication self-management by patients during hospitalisation.

Results of the nurse assessment are shown in Figure 1. Most patients were considered physically and mentally capable by nurses for in-hospital medication self-management, respectively, 75% and 78%. The statements that nurses most often disagreed with were related to the patient's capability to administer and prepare medication at home after discharge and the ability of patients to manage treatment, respectively, in 28% and 26% of the cases.

**Figure 2:**
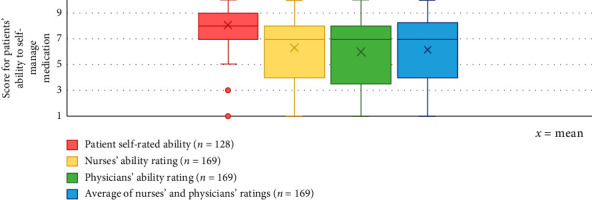
The extent to which a patient is considered capable of medication self-management during hospitalisation.

The patients' self-rated ability to self-manage medication in hospital was on average higher (mean score 8/10 [SD 1.57]) compared to the nurses' rating (mean score 6.3/10 [SD 2.71]) and physicians' rating (mean score 6/10 [SD 2.93]) (Figure 2).

However, the patients' self-rated ability to self-manage medication during hospitalisation correlated significantly with the nurses' rating (Spearman's *r* = 0.425, *p* < 0.001) and with the physicians' rating (Spearman's *r* = 0.383, *p* < 0.001). Furthermore, there was a strong relationship between the rating of the nurse and physician regarding the patient's ability to self-manage (Pearson's *r* = 0.821, *p* < 0.001).

### 3.4. Relationship Between Medication Literacy and Medication Self-Management

**Figure 3:**
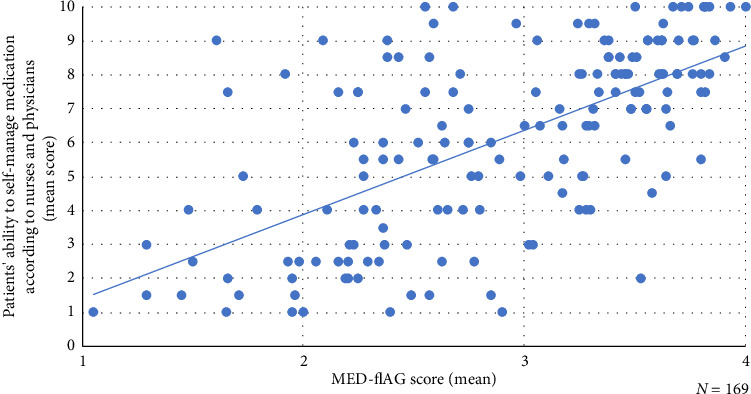
Scatterplot of the relationship between medication literacy skills (MED-fLAG) and patients' medication self-management capacity.

There was a strong positive correlation between patients' medication literacy skills and the extent to which patients are considered capable to self-manage their medication during hospitalisation according to nurses and physicians (Pearson's *r* = 0.639, *p* < 0.001) (Figure 3). Patients' ability for medication self-management most strongly correlated with the functional medication literacy domain (Pearson's *r* = 0.630, *p* < 0.001), followed by correlation with the interactive domain (Pearson's *r* = 0.590, *p* < 0.001) and the critical domain (Pearson's *r* = 0.582, *r* < 0.001). The more medication literacy skills showed by patients completing the MED-fLAG, the more patients were considered capable of self-management of medication as estimated by healthcare providers using SelfMED.

### 3.5. Factors Influencing the Strength of the Relationship Between MED-fLAG and SelfMED

Table 3 shows that 48.2% of participants fell in the same quartile for both SelfMED and MED-fLAG. For 39.6% of participants, SelfMED results are located one quartile higher or lower than MED-fLAG results. Moderation analysis could not identify any factors that significantly influenced the strength of the relationship between MED-fLAG and SelfMED. Age, education, gender, living situation, type of hospitalisation, the number of medications and the number of chronic conditions did not appear to be moderator variables.

### 3.6. Factors Influencing the Extent to Which Patients Are Considered Capable of Medication Self-Management

Detailed results are shown in Table 4. Significant differences in the extent to which patients were considered capable of medication self-management were observed. Higher scores were observed in patients who worked in health care (7.8/10 vs. 5.9/10, *p*=0.003), in patients who took 10 chronic medicines or less (6.4/10 vs. 5.2/10, *p*=0.013) and in participants who self-managed medication completely independent at home (7.3/10 vs. 3.8/10, *p* < 0.001). Furthermore, higher scores regarding medication self-management capacity were associated with lower age (*r* = −0.335, *p* < 0.001), less chronic medicines (*r* = −0.254, *p* < 0.001) and higher medication literacy skills (*r* = 0.639, *p* < 0.001). The multiple linear regression model explained 57.1% of the variance in the patients' medication self-management capacity score with medication self-management at home, age, number of chronic conditions and MED-fLAG scores as significant predictors.

## 4. Discussion

The first step to guide and support patients towards appropriate medication self-management is to assess the extent to which a patient is capable to self-manage medication [22, 23]. Medication literacy is, thereby, considered an important aspect for the performance of proper medication self-management. The main goal of this study was to explore the relationship between medication literacy and medication self-management. Findings suggest higher scores in interactive medication literacy, than in the functional and critical domains. For medication self-management, patients considered themselves more capable of in-hospital medication self-management than what perceived nurses and physicians. Finally, a strong positive association between medication literacy and medication self-management was found; the more medication literacy skills, the more patients were considered capable of medication self-management by healthcare providers. Age, managing medication independently at home, the number of chronic conditions and medication literacy were significant predictors of a patient's capability for in-hospital medication self-management.

Medication literacy was explored using the recently developed MED-fLAG [20]. Since no previous studies published assessment of medication literacy with this scale, it is therefore difficult to compare our findings with other study populations. Nevertheless, the functional domain of MED-fLAG covers the basic knowledge about medication allowing patients to prepare, self-administer and monitor the effects of their therapy [20]. In this regard, difficulties reported by the study participants are comparable to previous findings, showing substantial knowledge deficits in older patients concerning their medication. Congruent with our study, these include failing to recall the medication names, the reasons, the dosages, the times of administration, the treatment durations, the side effects and the precautions [30–38].

Interactive medication literacy had the highest score. In contrast to several other studies [39, 40], participants in the current study reported low difficulties to actively interact with healthcare providers and providing medication-related information (i.e., problems, preferences).

The critical domain of medication literacy, which refers to the strategies initiated to keep control over the medication management and the actualisation of medication-related knowledge from reliable sources of information, had the lowest score. The critical domain of medication literacy encompasses the degree to which patients can update medication-related knowledge from reliable sources [20]. In this regard, the package insert is the officially approved document to provide relevant and reliable information about the medication. However, participants in the current study indicate to not read the package insert often, which is congruent with previous research [41]. In today's digital era, people are using the Internet to access health-related information and make decisions [42–44]. With the perspective to support patients in using online medication-related information, healthcare professionals should consider supporting the patients to find quality and reliable online information, particularly among patients who show low digital health literacy [43, 45].

The extent to which patients were considered capable of in-hospital medication self-management was explored using SelfMED. In the SelfMED self-assessment, a patients' motivation is one of the elements taken into account when considering the ability to self-manage medication [46]. About 60% of patients would be willing to self-manage medication whilst in hospital. However, a previous study in hospitalised patients showed a higher willingness rate of 84% [46]. This may be due to a different study population: The current study focussed specifically on the hospitalised elderly with polypharmacy and not on the hospitalised general population.

It is noteworthy that patients considered themselves more capable of medication self-management compared to healthcare providers' assessment. We have to wonder whether patients overestimate their own ability to self-manage medication as several studies already showed that patients often encounter problems with medication management [5–8, 47, 48]. This emphasises that medication self-management assessment tools should not rely solely on patient self-assessments and highlights the added value of a stepped assessment that includes healthcare providers' perspectives (i.e., SelfMED).

The current study showed that the more medication literacy skills patients have according to the MED-fLAG, the more patients are considered able to self-manage medication, assessed by SelfMED, or vice versa. Thus, this study confirmed our hypothesis that there is a relation between both scores. Given the rather strong relationship between the two scores, it seems that SelfMED can be used as the first screening instrument to determine a patient's eligibility for in-hospital medication self-management, without first assessing medication literacy. However, if patients are considered less capable of medication self-management, the MED-fLAG can be of added value to provide more insight into medication literacy skills. Insight into these skills can help healthcare providers better tailor medication-related information and interventions to each patient's level.

SelfMED has the advantage of being a brief instrument, whereby the administrative burden for patients and nurses remains limited (i.e., filling out only 10 statements). Using only one instrument to determine patient's eligibility for medication self-management, rather than two, is more feasible in clinical practice and research, considering the current workload, staff shortages and time constraints. Our study demonstrated that that the strength of the relationship between MED-fLAG and SelfMED is not affected by sociodemographic factors such as age, gender, education, etc. Therefore, it is not possible to tailor the additional administration of MED-fLAG to specific target groups based on these results.

This study showed that patients with excessive polypharmacy (taking > 10 medicines) are considered less able to self-manage medication, compared to those who are taking less than 10 medicines. The complexity of the medication regimen associated with excessive polypharmacy probably contributes to this [49]. Assessing, monitoring and supporting medication management in this group of patients should be, therefore, considered as a priority.

Age seems to be an influencing factor of the extent to which patients are considered able to self-manage medication. Previous research already showed that higher age is associated with lower self-management capacity and lower health literacy [8, 50]. Furthermore, independent medication self-management at home appears to significantly predict being considered capable of in-hospital medication self-management. This result seems logic since patients who manage their medicines themselves are assumed to have better skills compared to patients who already receive support and guidance at home. In addition, as medication literacy is a predictor of the extent to which patients are considered capable of medication self-management, the study proves that medication literacy is an important element of medication self-management.

### 4.1. Strengths and Limitations

A first strength of the study was the high participation rate of 88%. The impact of participation bias is, therefore, rather small. Second, a heterogeneous sample of patients with different profiles was obtained due to the inclusion of patients on five different types of hospital wards, which benefits the generalisability of the results. Thirdly, the SelfMED assessment incorporates not only the patient's perspective to evaluate the extent to which a patient is capable to self-manage medication but also the perspective of the nurse and treating physician, which is likely to be more objective.

Nevertheless, there are some limitations. MED-fLAG is a patient-reported outcome measure which may be subject to desirability bias. However, further psychometric studies are needed to evaluate the measurement properties of MED-fLAG and to reduce the number of items before considering its clinical application. The SelfMED assessment aims to assess the extent to which patients are considered capable of self-management of medication whilst in hospital but does not include a formal assessment of medication management skills as such.

### 4.2. Implications for Nursing Management

The results in the current study may provide information for planning interventions, offering nurse managers an opportunity to improve the nursing discharge process, previously described as inconsistent [51, 52]. Congruent with previous research, our findings suggest that integration of medication intake into the daily routine could be challenging [6], including maintaining correct medication intake after prescription changes [53–55].

Nurse managers could improve the nursing discharge process by integrating systematic assessment of the difficulties encountered with medication management at home [7, 56], and providing individualised interventions (i.e., educational strategies, simplification of medication prescribing and medication aids) [57, 58]. In this regard, nurse managers should consider implementing in-hospital medication self-management, as it can be a promising strategy for patients to become acquainted with medication that is newly prescribed or altered, which probably will benefit medication knowledge [59]. The SelfMED assessment and the MED-fLAG are valuable tools to support these interventions.

## 5. Conclusion

This study showed a significant positive relationship between medication literacy, measured by the MED-fLAG, and the extent to which patients are considered capable to self-manage medication in hospital, measured by the SelfMED assessment. This correlation implies that the SelfMED assessment is appropriate for assessing patients' eligibility for medication self-management during hospitalisation, without first explicitly assessing medication literacy skills as an important aspect of medication self-management. MED-fLAG is very valuable to provide additional insight in order to tailor medication-related information and interventions to the patients' medication literacy level.

## Figures and Tables

**Table 1:** Sample characteristics (*N* = 169).

Characteristics		*n* (%)
Gender	Male	97 (57.4)
	Female	72 (42.6)

Highest education level (EQF⁣^∗^)	EQF Level 1	21 (12.4)
	EQF Level 2/3/4	65 (38.5)
	EQF Level 5/6	82 (48.5)
	EQF Level 7	1 (0.6)

Living alone (yes)		57 (33.7)

Help needed to reside at home	Yes	107 (63.3)

Type of hospitalisation	Planned	52 (30.8)
	Unplanned	117 (69.2)

Reason for hospitalisation	Treatment	83 (49.1)
	Observation	28 (16.6)
	Surgery	24 (14.2)
	Examination	24 (14.2)
	Medication review	10 (5.9)

Type of hospital ward	Oncology	59 (35.0)
	Cardiology	39 (23.1)
	Geriatrics	31 (18.3)
	Surgical ward	25 (14.8)
	Internal medicine	14 (8.3)

Length of hospital stay	2-3 days	85 (50.3)
	4–7 days	54 (32.0)
	> 7 days	30 (17.8)

Number of medicines per day	< 5 medicines	2 (1.2)
	5–10 medicines	129 (76.3)
	> 10 medicines	36 (21.3)
	Don't know	2 (1.2)

		**Median (min–max; IQR)**

Age		76 (65–101; 9)
Number of chronic conditions		2 (0–5; 1)

⁣^∗^European Qualifications Framework [26]; Level 1: primary education, Level 2: lower secondary education, Level 3: upper secondary vocational education, Level 4: upper secondary education, Level 5: associate's degree, Level 6: bachelor's degree, Level 7: master's degree.

**Table 2:** Patient self-assessment (*N* = 169).

				%
Medication management at home	Completely independent			66.9
	Help with organisation and preparation, independent administration			30.1
	Help with organisation, preparation and administration			3.0

Willingness to self-manage medication in hospital (*n* = 167)	Willing			58.1
	Not willing			41.9

				**Mean ± SD [range]**

Self-rated ability to self-manage medication in hospital (*n* = 128)				8.1 ± 1.57 [1–10]

	**%**			

Statements regarding medication adherence (*n* = 128)	Never	Sometimes	Often	Always
Forgot to take medicines	76.6	23.4	0.0	0.0
Not taking medicines for other reasons than forgetting	82.0	18.0	0.0	0.0
Stopped taking medicines without reporting to a physician	89.8	9.4	0.8	0.0
Not taking medicines on vacation	92.2	7.0	0.8	0.0
Stopped medicines' use when feeling better	86.7	11.7	1.6	0.0
Difficulties to stick to the treatment schedule	90.6	9.4	0.0	0.0
Difficulties to remember medication taken	89.1	10.9	0.0	0.0

**Table 3:** Percentage of patients classified by MED-fLAG and SelfMED quartiles (*N* = 169).

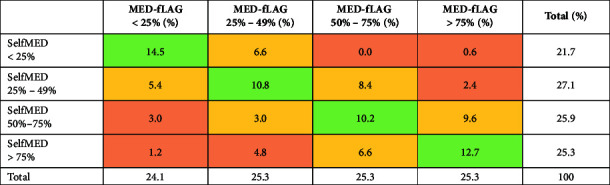

*Note:* 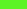: results fall within the same quartile; 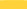: results differ by one quartile; 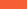: results differ by more than one quartile.

**Table 4:** Influencing factors of the extent to which patients are considered capable of medication self-management (*N* = 169).

Variables	Descriptive analysis		Univariate linear regression			Multiple linear regression (*n* = 169, adjusted *R*^2^ = 0.571, *p* < 0.001)				
	Mean score medication self-management capacity (…/10) ⁣^∗^	Independent *t* test *p* value	*B*	Bèta	*p* value	*B*	Bèta	CI 95% B (lower)	CI 95% B (upper)	*p* value
Gender										
Male (*n* = 97)	6.2	0.425	−0.052	−0.010	0.901					
Female (*n* = 72)—ref.	6.1									
Education										
≥ than EQF Level 5 (*n* = 83)	6.0	0.541	−0.255	−0.048	0.539					
< than EQF Level 5 (*n* = 86)—ref.	6.3									
Worked in health care										
Yes (*n* = 19)	7.8	**0.003**	1.909	0.225	**0.003**					
No (*n* = 150)—ref.	5.9									
Living situation										
Cohabitation (*n* = 112)	6.1	0.877	−0.068	−0.012	0.877					
Alone (*n* = 57)—ref.	6.2									
Help needed to reside at home										
Yes (*n* = 107)	5.5	**< 0.001**	−1.690	−0.304	**< 0.001**					
No (*n* = 62)—ref.	7.2									
Type of hospitalisation										
Unplanned (*n* = 117)	6.2	0.820	0.103	0.018	0.820					
Planned (*n* = 52)—ref.	6.1									
Reason for hospitalisation										
Medication review (*n* = 10)	6.9	0.364	0.799	0.070	0.364					
Other reason (*n* = 159)—ref.	6.1									
Number of chronic medicines										
≤ 10 medicines (*n* = 131)	6.4	**0.013**	−1.241	−0.191	**0.013**					
> 10 medicines (*n* = 36)	5.2									
Medication self-management at home										
Completely independent (*n* = 113)	7.3	**< 0.001**	3.466	0.608	**< 0.001**	1.667	0.293	0.965	2.369	**< 0.001**
Help with preparing and/or administering (*n* = 56)—ref.	3.8									

	**Spearman's rho correlation**	**p** **-value**								

Age (*n* = 169)	−0.335	**< 0.001**	−0.134	−0.366	**< 0.001**	−0.075	−0.205	−0.114	−0.036	**< 0.001**
Number of chronic conditions (*n* = 169)	−0.254	**< 0.001**	−0.683	−0.278	**< 0.001**	−0.532	−0.217	−0.794	−0.270	**< 0.001**

	**Pearson's correlation**	**p** **-value**								

Mean medication literacy score [1–4] (*n* = 169)	0.639	**< 0.001**	2.479	0.639	**< 0.001**	1.626	0.419	1.155	2.097	**< 0.001**

*Note:* The bold values are statistically significant.

⁣^∗^Mean score of the physician's and nurse's ratings of a patient's medication self-management capacity.

## Data Availability

The data used to support the findings of this study are available from the corresponding author upon reasonable request.

## Supporting Information

Supporting Table S1: Mean item scores categorised by medication literacy domain.

## References

[1] Eurostat (2023). Population Structure and Ageing. https://ec.europa.eu/eurostat/statistics-explained/index.php?title=Population_structure_and_ageing.

[2] Aggarwal P., Woolford S. J., Patel H. P. (2020). Multi-Morbidity and Polypharmacy in Older People: Challenges and Opportunities for Clinical Practice. *Geriatrics*.

[3] Grady P. A., Gough L. L. (2014). Self-Management: A Comprehensive Approach to Management of Chronic Conditions. *American Journal of Public Health*.

[4] Pulvirenti M., McMillan J., Lawn S. (2014). Empowerment, Patient Centred Care and Self-Management. *Health Expectations*.

[5] Bailey S. C., Oramasionwu C. U., Wolf M. S. (2013). Rethinking Adherence: A Health Literacy-Informed Model of Medication Self-Management. *Journal of Health Communication*.

[6] Dijkstra N. E., Sino C. G. M., Schuurmans M. J., Schoonhoven L., Heerdink E. R. (2020). Medication Self-Management: Considerations and Decisions by Older People Living at Home. *Research in Social and Administrative Pharmacy*.

[7] Mortelmans L., De Baetselier E., Goossens E., Dilles T. (2021). What Happens after Hospital Discharge? Deficiencies in Medication Management Encountered by Geriatric Patients With Polypharmacy. *International Journal of Environmental Research and Public Health*.

[8] Sino C. G., Sietzema M., Egberts T. C., Schuurmans M. J. (2014). Medication Management Capacity in Relation to Cognition and Self-Management Skills in Older People on Polypharmacy. *The Journal of Nutrition, Health & Aging*.

[9] Barnett K., Mercer S. W., Norbury M., Watt G., Wyke S., Guthrie B. (2012). Epidemiology of Multimorbidity and Implications for Health Care, Research, and Medical Education: A Cross-Sectional Study. *Lancet*.

[10] Corsonello A., Pedone C., Incalzi R. A. (2010). Age-related Pharmacokinetic and Pharmacodynamic Changes and Related Risk of Adverse Drug Reactions. *Current Medicinal Chemistry*.

[11] van den Akker M., Vaes B., Goderis G., Van Pottelbergh G., De Burghgraeve T., Henrard S. (2019). Trends in Multimorbidity and Polypharmacy in the Flemish-Belgian Population Between 2000 and 2015. *PLoS One*.

[12] Fried T. R., O’Leary J., Towle V., Goldstein M. K., Trentalange M., Martin D. K. (2014). Health Outcomes Associated With Polypharmacy in Community-Dwelling Older Adults: A Systematic Review. *Journal of the American Geriatrics Society*.

[13] Harris C. M., Sridharan A., Landis R., Howell E., Wright S. (2013). What Happens to the Medication Regimens of Older Adults During and After an Acute Hospitalization?. *Journal of Patient Safety*.

[14] Bagge M., Norris P., Heydon S., Tordoff J. (2014). Older People’s Experiences of Medicine Changes on Leaving Hospital. *Research in Social and Administrative Pharmacy*.

[15] Persell S. D., Karmali K. N., Lee J. Y. (2020). Associations Between Health Literacy and Medication Self-Management Among Community Health Center Patients With Uncontrolled Hypertension. *Patient Preference and Adherence*.

[16] Kripalani S., Henderson L. E., Chiu E. Y., Robertson R., Kolm P., Jacobson T. A. (2006). Predictors of Medication Self-Management Skill in a Low-Literacy Population. *Journal of General Internal Medicine*.

[17] Davis T. C., Wolf M. S., Bass P. F. (2006). Literacy and Misunderstanding Prescription Drug Labels. *Annals of Internal Medicine*.

[18] Bazaldua O. V., Davidson D. A., Zurek A., Kripalani S., DiPiro J. T. (2017). Health Literacy and Medication Use. *Pharmacotherapy: A Pathophysiologic Approach, 10e*.

[19] Mackey L. M., Doody C., Werner E. L., Fullen B. (2016). Self-Management Skills in Chronic Disease Management: What Role Does Health Literacy Have?. *Medical Decision Making*.

[20] Gentizon J., Fleury M., Pilet E., Büla C., Mabire C. (2022). Conceptualization and Content Validation of the MEDication Literacy Assessment of Geriatric Patients and Informal Caregivers (MED-fLAG). *Journal of Patient-Reported Outcomes*.

[21] Gentizon J., Bovet E., Rapp E., Mabire C. (2022). Medication Literacy in Hospitalized Older Adults: Concept Development. *Health Literacy Research and Practice*.

[22] Advinha A. M., Lopes M. J., de Oliveira-Martins S. (2017). Assessment of the Elderly’s Functional Ability to Manage Their Medication: A Systematic Literature Review. *International Journal of Clinical Pharmacy*.

[23] Badawoud A. M., Salgado T. M., Lu J., Parsons P., Peron E. P., Slattum P. W. (2020). Measuring Medication Self-Management Capacity: A Scoping Review of Available Instruments. *Drugs & Aging*.

[24] Vanwesemael T., Dilles T., Van Rompaey B., Boussery K. (2018). An Evidence-Based Procedure for Self-Management of Medication in Hospital: Development and Validation of the SelfMED Procedure. *Pharmacy*.

[25] Tabachnick B. G., Fidell L. S. (2012). *Using Multivariate Statistics*.

[26] European Centre for the Development of Vocational Training European Qualifications Framework (EQF). https://www.cedefop.europa.eu/en/events-and-projects/projects/european-qualifications-framework-eqf.

[27] Vanwesemael T., Mortelmans L., Boussery K., Jordan S., Dilles T. (2022). Self-Management of Medication on a Cardiology Ward: Feasibility and Safety of the SelfMED Intervention. *International Journal of Environmental Research and Public Health*.

[28] Kim H. Y. (2013). Statistical Notes for Clinical Researchers: Assessing Normal Distribution (2) Using Skewness and Kurtosis. *Restorative Dentistry & Endodontics*.

[29] World Medical Association (2013). World Medical Association Declaration of Helsinki: Ethical Principles for Medical Research Involving Human Subjects. *JAMA*.

[30] Passagli L. C., Barros Cota B., César Simões T., Chama Borges Luz T. (2021). Knowledge of Prescribed Drugs Among Primary Care Patients: Findings From Prover Project. *International Journal of Clinical Pharmacy*.

[31] Bosch-Lenders D., Maessen D. W., Stoffers H. E., Knottnerus J. A., Winkens B., van den Akker M. (2016). Factors Associated With Appropriate Knowledge of the Indications for Prescribed Drugs Among Community-Dwelling Older Patients With Polypharmacy. *Age and Ageing*.

[32] Ramia E., Zeenny R. M., Hallit S., Salameh P. (2017). Assessment of Patients’ Knowledge and Practices Regarding Their Medication Use and Risks in Lebanon. *International Journal of Clinical Pharmacy*.

[33] Najjar A., Amro Y., Kitaneh I. (2015). Knowledge and Adherence to Medications Among Palestinian Geriatrics Living With Chronic Diseases in the West Bank and East Jerusalem. *PLoS One*.

[34] Okuyan B., Sancar M., Izzettin F. V. (2013). Assessment of Medication Knowledge and Adherence Among Patients under Oral Chronic Medication Treatment in Community Pharmacy Settings. *Pharmacoepidemiology and Drug Safety*.

[35] Rubio J. S., García-Delgado P., Iglésias-Ferreira P., Mateus-Santos H., Martínez-Martínez F. (2015). Measurement of Patients’ Knowledge of Their Medication in Community Pharmacies in Portugal. *Ciência & Saúde Coletiva*.

[36] Bulut H., Tanrıkulu G., Dal Ü., Kapucu S. (2013). How Much Do ED Patients Know About Medication Prescribed for Them on Discharge? A Pilot Study in Turkey. *Journal of Emergency Nursing*.

[37] Cumbler E., Wald H., Kutner J. (2010). Lack of Patient Knowledge Regarding Hospital Medications. *Journal of Hospital Medicine*.

[38] Zhong Z., Zheng F., Guo Y., Luo A. (2016). Medication Literacy in a Cohort of Chinese Patients Discharged With Acute Coronary Syndrome. *International Journal of Environmental Research and Public Health*.

[39] Ozavci G., Bucknall T., Woodward-Kron R. (2021). A Systematic Review of Older Patients’ Experiences and Perceptions of Communication About Managing Medication across Transitions of Care. *Research in Social and Administrative Pharmacy*.

[40] Stevenson F. A., Cox K., Britten N., Dundar Y. (2004). A Systematic Review of the Research on Communication Between Patients and Health Care Professionals About Medicines: The Consequences for Concordance. *Health Expectations*.

[41] Bhosale U. A. (2016). Evaluation of Knowledge and Awareness of Patients About Prescribed Drugs and Their Package Inserts: A Cross-Sectional Study. *Asian Journal of Pharmaceutics*.

[42] Kummervold P., Chronaki C., Lausen B. (2008). eHealth Trends in Europe 2005–2007: A Population-Based Survey. *Journal of Medical Internet Research*.

[43] Hämeen-Anttila K., Pietilä K., Pylkkänen L., Pohjanoksa-Mäntylä M. (2018). Internet as a Source of Medicines Information (MI) Among Frequent Internet Users. *Research in Social and Administrative Pharmacy*.

[44] Bergmo T. S., Sandsdalen V., Manskow U. S., Småbrekke L., Waaseth M. (2023). Internet Use for Obtaining Medicine Information: Cross-Sectional Survey. *JMIR Formative Research*.

[45] Arias López M., Ong B. A., Borrat Frigola X. (2023). Digital Literacy as a New Determinant of Health: A Scoping Review. *PLoS Digital Health*.

[46] Vanwesemael T., Boussery K., van den Bemt P., Dilles T. (2018). The Willingness and Attitude of Patients towards Self-Administration of Medication in Hospital. *Therapeutic Advances in Drug Safety*.

[47] Notenboom K., Beers E., van Riet-Nales D. A. (2014). Practical Problems With Medication Use that Older People Experience: A Qualitative Study. *Journal of the American Geriatrics Society*.

[48] Mira J. J., Lorenzo S., Guilabert M., Navarro I., Pérez-Jover V. (2015). A Systematic Review of Patient Medication Error on Self-Administering Medication at Home. *Expert Opinion on Drug Safety*.

[49] George J., Phun Y. T., Bailey M. J., Kong D. C., Stewart K. (2004). Development and Validation of the Medication Regimen Complexity Index. *The Annals of Pharmacotherapy*.

[50] Plaza-Zamora J., Legaz I., Osuna E., Pérez-Cárceles M. D. (2020). Age and Education as Factors Associated With Medication Literacy: A Community Pharmacy Perspective. *BMC Geriatrics*.

[51] Hayajneh A. A., Hweidi I. M., Abu Dieh M. W. (2020). Nurses’ Knowledge, Perception and Practice Toward Discharge Planning in Acute Care Settings: A Systematic Review. *Nursing Open*.

[52] Mabire C., Bula C., Morin D., Goulet C. (2015). Nursing Discharge Planning for Older Medical Inpatients in Switzerland: A Cross-Sectional Study. *Geriatric Nursing*.

[53] Ziaeian B., Araujo K. L., Van Ness P. H., Horwitz L. I. (2012). Medication Reconciliation Accuracy and Patient Understanding of Intended Medication Changes on Hospital Discharge. *Journal of General Internal Medicine*.

[54] Pasina L., Brucato A. L., Falcone C. (2014). Medication Non-adherence Among Elderly Patients Newly Discharged and Receiving Polypharmacy. *Drugs & Aging*.

[55] Schoonover H., Corbett C. F., Weeks D. L., Willson M. N., Setter S. M. (2014). Predicting Potential Postdischarge Adverse Drug Events and 30-Day Unplanned Hospital Readmissions From Medication Regimen Complexity. *Journal of Patient Safety*.

[56] Daliri S., Bekker C. L., Buurman B. M., Scholte Op Reimer W. J. M., van den Bemt B. J. F., Karapinar-Carkit F. (2019). Barriers and Facilitators With Medication Use During the Transition From Hospital to Home: A Qualitative Study Among Patients. *BMC Health Services Research*.

[57] Ryan R., Santesso N., Lowe D. (2014). Interventions to Improve Safe and Effective Medicines Use by Consumers: An Overview of Systematic Reviews. *Cochrane Database of Systematic Reviews*.

[58] Cross A. J., Elliott R. A., Petrie K., Kuruvilla L., George J. (2020). Interventions for Improving Medication-Taking Ability and Adherence in Older Adults Prescribed Multiple Medications. *Cochrane Database of Systematic Reviews*.

[59] Vanwesemael T., Boussery K., Manias E., Petrovic M., Fraeyman J., Dilles T. (2018). Self-Management of Medication During Hospitalisation: Healthcare Providers’ and Patients’ Perspectives. *Journal of Clinical Nursing*.

[60] Vanwesemael T. (2018). The Attitude and Opinion of Healthcare Providers on Self-Administration of Medication by Hospitalized Patients. *SelfMED. Patient Self-Management of Medication in Hospital*.

