# Supplementary material for: Medication Literacy and Medication Self-Management: A Cross-Sectional Study in Hospitalised Patients (65+) With Polypharmacy
Source: J Nurs Manag. 2024 Dec 31;2024:5430265. doi: 10.1155/jonm/5430265 (PMC11922036; doi:10.1155/jonm/5430265)
Supplement: Supporting Information — Additional supporting information can be found online in the Supporting Information section. [file 5430265.f1.docx]

**Supplementary Table S1: Mean item scores categorised by medication literacy domain.**

|  | **Description of each item** | **Mean [SD]*** |
| --- | --- | --- |
| **Interactive medication literacy (IML)** | Providing information about known allergies or poorly tolerated drugs (IML 30) | 3.3 [0.99] |
|  | Asking health professionals for additional information about your medicines (IML 23) | 3.3 [0.86] |
|  | Discussing any difficulties in following the medication plan or following instructions (IML 31) | 3.2 [0.90] |
|  | Reporting about mistakes, omissions or other problems with medication (IML 32) | 3.2 [0.93] |
|  | Telling a health professional if you have stopped or changes the dosage of a medicine (IML 33) | 3.2 [0.95] |
|  | Expressing your expectations; giving you opinion about medicines (IML 27) | 3.1 [0.97] |
|  | Asking health professionals for advice about natural remedies (IML 24) | 3.1 [1.00] |
|  | Understanding information about medicines given by health professionals (IML 25) | 3.0 [1.05] |
|  | Seeking professional advice about information about medicines obtained from the media, social networks, … (IML 35) | 3.0 [1.08] |
|  | Talking about conflicting information you have received about your medicines (IML 34) | 2.9 [1.09] |
|  | Describing the latest changes that have been made in the list of medicines (IML 26) | 2.8 [1.15] |
|  | Providing information about the effects observed or experienced (IML 29) | 2.6 [0.99] |
|  | Expressing your interest in natural remedies (IML 28) | 2.6 [1.09] |
|  | **Across all items of the functional domain** | 3.0 [0.79] |
| **Functional medication literacy (FML)** | Telling how/where to store medicines (FML 5) | 3.3 [0.99] |
|  | Telling which medicine should be taken before, during or after a meal (FML 11) | 3.2 [0.98] |
|  | Telling the schedule (hours) for taking each medicine (FML 12) | 3.2 [0.98] |
|  | Telling whether any of your medicines require special precautions (FML 22) | 3.2 [1.09] |
|  | Telling which medicines are pills, drops, inhalations,… (FML 4) | 3.1 [1.00] |
|  | Saying how long each medicine should be taken (FML 14) | 3.1 [1.06] |
|  | Telling whether certain foods or drinks should be avoided (FML 21) | 3.1 [1.06] |
|  | Saying whether the list of current medicines included PRN medicines (FML 10) | 3.1 [1.09] |
|  | Saying how many times a day each medicine should be taken (FML 13) | 3.1 [1.08] |
|  | Counting how many medicines need to be prepared to cover several days of treatment (FML 17) | 3.1 [1.06] |
|  | Preparing the correct dosage of medicines (FML 16) | 3.1 [1.03] |
|  | Describing medicines by their appearance (colour and shape) (FML 3) | 3.1 [1.01] |
|  | Telling whether any of the medicines may have effects that require monitoring (FML 19) | 3.0 [1.08] |
|  | Explaining what effect each medicines is expected to have (FML 8) | 3.0 [1.04] |
|  | Explaining why medicines has been prescribed (FML 7) | 3.0 [1.07] |
|  | Describing the problems that might occur if the medicines are not taken/taken differently than instructed (FML 9) | 2.8 [0.98] |
|  | Giving the names of the various prescribers of the medicines you manage (FML 6) | 2.6 [1.08] |
|  | Listing the names of natural remedies you manage (FML 2) | 2.6 [1.01] |
|  | Listing the names of medicines you manage (FML 1) | 2.5 [1.04] |
|  | Telling whether certain medicines are incompatible with the medicine you are managing (FML 20) | 2.3 [1.09] |
|  | Telling the dosage of each medicine by heart (FML 15) | 2.2 [1.15] |
|  | Listing the main side effects of medicines (FML 18) | 2.2 [1.05] |
|  | **Across all items of the functional domain** | 2.9 [0.76] |
| **Critical medication literacy (CML)** | Setting up daily routines to avoid forgetting to take medicines (CML 49) | 3.3 [0.93] |
|  | Going to the same pharmacy to get medicines (CML 48) | 3.3 [0.99] |
|  | Organising stock of medicines and knowing when to go to the pharmacy to get a refill or to get new medicines (CML 51) | 3.3 [0.87] |
|  | Adapting daily routines after the list of medicines has changed (CML 52) | 3.2 [0.92] |
|  | Knowing which healthcare professional to contact in case of problems with medicines (CML 56) | 3.2 [0.95] |
|  | Getting answers to questions about medicines, obtaining advice (CML 53) | 3.2 [0.96] |
|  | Knowing when to schedule a prescription renewal (CML 50) | 3.2 [1.03] |
|  | Getting help from health professionals in case of problems with medicines (CML 55) | 3.1 [0.92] |
|  | Getting help from you family in case of difficulties with medicines (CML 54) | 3.1 [1.03] |
|  | Writing down questions about medicines for health professionals (CML 46) | 2.8 [1.08] |
|  | Carrying a list of the medicines you manage (CML 41) | 2.8 [1.20] |
|  | Reading patient leaflets about medicines (CML 37) | 2.8 [1.04] |
|  | Using a routine, some tricks that allow to check that the medicines have not been forgotten (CML 43) | 2.7 [1.18] |
|  | Reading the package leaflet (CML 36) | 2.6 [1.09] |
|  | Questioning the reliability of information about medicines obtained from the media, social networks,… (CML 40) | 2.6 [0.98] |
|  | Taking some medicines with you when you leave home (CML 45) | 2.6 [1.15] |
|  | Using a treatment plan that describes the medicines that need to be taken (CML 42) | 2.6 [1.17] |
|  | Keeping a record of the decisions that have been made about medicines (CML 47) | 2.5 [1.17] |
|  | Seeking advice from a health professional before taking any over-the-counter medicines (CML 38) | 2.4 [1.01] |
|  | Using a pillbox (CML 44) | 2.3 [1.23] |
|  | Seeking information when a new medicines has been prescribed (CML 39) | 2.2 [1.18] |
|  | **Across all items of the critical domain** | 2.8 [0.68] |
| * Mean score of each item on a four-point Likert scale (1= very difficult/never; 4 = not difficult at all/always). The higher the score, the more medication literacy skills. | | |
